# A hybrid planning strategy for stereotactic body radiation therapy of early stage non‐small‐cell lung cancer

**DOI:** 10.1002/acm2.12450

**Published:** 2018-10-03

**Authors:** Han Liu, Benjamin Sintay, Keith Pearman, Qingyang Shang, Lane Hayes, Jacqueline Maurer, Caroline Vanderstraeten, David Wiant

**Affiliations:** ^1^ Department of Radiation Oncology Cone Health Cancer Center Greensboro NC USA

**Keywords:** DCA, hybrid, lung SBRT, VMAT

## Abstract

Currently dynamic conformal arcs (DCA) and volumetric modulated arc therapy (VMAT) are two popular planning techniques to treat lung stereotactic body radiation therapy (SBRT) patients. Of the two, DCA has advantages in terms of multi‐leaf collimator (MLC) motion, positioning error, and delivery efficiency. However, VMAT is often the choice when critical organ sparing becomes important. We developed a hybrid strategy to incorporate DCA component into VMAT planning, results were compared with DCA and VMAT plans. Four planning techniques were retrospectively simulated for 10 lung SBRT patients: DCA, Hybrid‐DCA (2/3 of the doses from DCA beams), Hybrid‐VMAT (2/3 of the doses from VMAT beams) and VMAT. Plan complexity was accessed by modulation complexity score (MCS). Conformity index (CI) for the planning target volume (PTV), *V*_20_ and *V*_5_ for the lung, *V*_30_ for the chestwall, and maximum dose to all other critical organs were calculated. Plans were compared with regard to these metrics and measured agreement between the planned and delivered doses. DCA technique did not result in acceptable plan quality due to target location for five patients. Hybrid‐DCA produced one unacceptable plan, and Hybrid‐VMAT and VMAT produced no unacceptable plans. The CI improved with increasing VMAT usage, as did the dose sparing to critical structures. Compared to the VMAT technique, a total MU reduction of 14%, 25% and 37% were found for Hybrid‐VMAT, Hybrid‐DCA and DCA techniques for 54 Gy patient group, and 9%, 23% and 34% for 50 Gy patient group, suggesting improvement in delivery efficiency with increasing DCA usage. No significant variations of plan complexity were observed between Hybrid‐DCA and Hybrid‐VMAT (*P *= 0.46 from Mann–Whitney *U*‐test), but significant differences were found among DCA, Hybrid and VMAT (*P* < 0.05). Better agreements between the planned and delivered doses were found with more DCA contributions. By adding DCA components to VMAT planning, hybrid technique offers comparable dosimetry to full VMAT, while increasing delivery efficiency and minimizing MLC complexity.

## 1 INTRODUCTION

Stereotactic body radiation therapy (SBRT) is an advanced technique that is becoming the treatment choice for medically inoperable and many high‐risk surgical non‐small‐cell lung cancer (NSCLC) patients. SBRT is able to precisely deliver high doses to tumors while sparing adjacent normal tissues in five or fewer treatment fractions. Compared to conventional radiation therapy treatment, SBRT offers superior outcomes, lower costs and greater patient convenience.^1^, ^2^, ^3^, ^4^, ^5^

Three‐dimensional conformal radiation therapy (3D‐CRT), dynamic conformal arcs (DCA), intensity modulated radiation therapy (IMRT), and volumetric modulated arc therapy (VMAT) have been used as delivery options for Linac‐based SBRT treatments. Recently DCA and VMAT have become more and more popular due to their delivery efficiency compared to 3D‐CRT and IMRT. DCA is a forward planning technique, where the planner defines arc geometries (gantry start/stop angles, couch and collimator angles, relative arc weightings, etc.) and the multi‐leaf collimator (MLC) are shaped to conform to the target in the beam's eye view (BEV) as the gantry rotates around the patient. The gantry speed and dose rate remain constant during the DCA delivery.

VMAT was first introduced and implemented into the clinic as a novel radiation delivery technique and a variation of static field IMRT.^6^, ^7^ By using an inverse planning algorithm, VMAT technique allows high modulation of the gantry rotation speed, dose rate, and the position and speed of MLC, to achieve highly conformal dose distributions around the target.

It is still a matter of debate which technique, DCA or VMAT, is superior for delivering SBRT to lung cancer patients.^8^ DCA may be favored over VMAT for the following reasons: (a) less MLC motion complexity (less susceptible to the interplay effect between tumor and MLC motion), (b) less MLC positon errors, (c) better delivery efficiency, (d) less affected by the accuracy of the small field dosimetry modeling in the treatment planning system, (e) more cost effective, and (f) the possibility of no patient specific QA measurements before the start of treatment, etc. However, VMAT may provide increased ability for dose shaping, which becomes important when target shape is irregular or the target is in proximity to certain critical structures.

Both DCA and VMAT techniques have their own advantages and disadvantages. In this study we explore adding partial MLC modulation to a DCA plan to increase the dose shaping around the target but maintain many of the advantages of 3D conformal beams. BrainLab (BrainLab AG, Feldkirchen, Germany) has developed a HybridArc strategy, which blends aperture‐enhanced optimized arcs with several static IMRT‐elements at specific intervals. By weighting the contribution of arcs vs IMRT, HybridArc is able to achieve an optimal dose distribution.^9^, ^10^ Instead of using IMRT beams, in this study we developed a hybrid planning strategy to incorporate DCA component into VMAT planning. The results of this technique are compared with DCA and VMAT plans in terms of plan quality, plan complexity, treatment delivery efficiency, and the agreement between the planned and delivered doses.

## 2 MATERIALS AND METHODS

Ten NSCLC patients (5 received 54 Gy in 3 fractions and 5 received 50 Gy in 5 fractions) treated with SBRT were retrospectively reviewed in this study. All patients were positioned supine and immobilized with a customized vacuum bag restriction system (Bodyfix, Medical Intelligence Inc) for simulation and subsequent treatments. Motion management of the tumor was achieved with a paddle‐based abdominal compression device. All patients underwent a free breathing and ten‐phase four‐dimensional computer tomography (4DCT) scan on a Philips Brilliance Big Bore CT scanner (Philips, Cleveland, OH). The Philips bellows system was placed around the abdomen to monitor the patient respiratory motion. The free breathing CTs were used for treatment planning and were acquired with fields of view large enough to cover the patient and immobilization devices with 2 mm slice thickness. An internal target volume (ITV) was generated based on the maximum intensity projection (MIP) of 4DCT image, and the planning target volume (PTV) was created by a 5 mm uniform expansion of the ITV. All patients were treated on a Varian TrueBeam STx platform with cone beam CT (CBCT) image guidance.

Four planning techniques were simulated in this study: DCA, Hybrid‐DCA (2/3 of the doses from DCA beams), Hybrid‐VMAT (2/3 of the doses from VMAT beams) and VMAT. The same beam configurations (gantry, collimator and table angles) were used for all four techniques. For the hybrid strategies, DCA beams were conformed to the PTV on the beams‐eye‐view to deliver a fraction (1/3 or 2/3) of the prescription dose. The doses from DCA beams were used as the base dose in the VMAT optimization, which was used to shape the dose distributions. For the same patient, half arcs with same start and stop gantry angles were used for all four techniques. The same planning objectives, constrains, and weighting for the target and critical organs were used for both hybrid and VMAT plans. The setting were also retained for normal tissue optimization (NTO) and the MU objectives. All plans used coplanar 6 MV flattening filter free (6X‐FFF) beams, and were normalized such that at least 95% of the PTV received the prescription dose, and more than 99% of the PTV received at least 90% of the prescription dose. PTV coverages were forced to be identical for the same patient in order to have a fair comparison between all different strategies.

The maximum point dose and dose‐volume constraints of several critical structures are listed in Table 1 for both 54 Gy and 50 Gy protocols.^11^, ^12^, ^13^ Selected dose‐volume parameters were compared, including the conformity index (*CI*_50_, ratio of the volume receiving 50% of prescription dose to the PTV volume), *V*_20_ and *V*_5_ (lung volumes receiving 20 Gy and higher, and 5 Gy and higher, respectively) for combined lungs, *V*_30 Gy_ (volume receiving 30 Gy and higher) for the chest wall, and *D*_0.035 cc_ (dose to 0.035 cc of the volume, a representative of maximum dose) for all other critical structures such as the spinal cord, aorta, trachea, etc.

**Table 1:** Lung SBRT planning acceptance objectives for critical structures

OARs	Volume	54 Gy/3 fractions		50 Gy/5 fractions	
		Threshold dose (Gy)	Max point dose (Gy)	Threshold dose (Gy)	Max point dose (Gy)
Spinal cord	<0.25 cc <0.5 cc		18	22.5 13.5	30
Lungs‐ITV	<15%	20		20	
Esophagus	<5 cc	17.7	25.2	19.5	35
Aorta	<10 cc	39	45	47	53
Trachea	<4 cc	15	30	16.5	40
Skin	<10 cc	30	33	36.5	39.5
Chest wall	<30 cc	30		30	

In this study, the plan complexity was assessed by the MCS, which was originally developed by McNiven et al. to evaluate plan complexity and deliverability of step‐and‐shoot IMRT plans,^14^ and later was modified by Masi et al. in order to apply for VMAT plans (considering control points of the arc instead of segments).^15^ By definition MCS has a value between 0 and 1, where a higher MCS value means less complex MLC delivery. The MCS analysis was implemented as a plug‐in script to the Varian Eclipse treatment planning system. The agreement of the planned and delivered doses was evaluated with gamma passing rates (*γ*) from patient specific QA. Patient specific QAs were performed with an ArcCHECK device (Sun Nuclear Corporation, Melbourne, FL, USA) and *γ* were compared between different planning strategies for the same patient. The criteria for gamma analysis are 3%/3 mm and 2%/2 mm with 10% threshold. The Mann–Whitney *U* test was used to evaluate the statistical significance of the MCS values among different planning strategies. A Pearson correlation test was performed to evaluate the correlation among the MCS, total MUs and gamma passing rate.

All work was carried out with the approval of the institutional review board under protocol number 1767.

## 3 RESULTS

The average PTV volume were 36.4 ± 12.3 cc for the 54 Gy patient group and 38.8 ± 15.4 cc for the 50 Gy patient group, respectively. Table 2 shows the tumor locations and target coverages included in this analysis.

**Table 2:** Patient characteristics and PTV coverage. LUL = left upper lobe; LLL = left lower lube; RUL = right upper lobe; RLL = right lower lube; RML = right middle lobe

Patient ID	54 Gy/3 fractions			50 Gy/5 fractions		
	*V*_PTV_ (cc)	Tumor Location	PTV coverage (%)	*V*_PTV_ (cc)	Tumor Location	PTV coverage (%)
1	19.6	LUL	97.0	19.1	LLL	97.0
2	54.2	LUL	96.0	35.3	RUL	97.0
3	34.2	RML	97.0	47.6	RLL	95.0
4	36.9	LUL	97.0	59.5	RLL	97.0
5	37.2	RUL	97.0	32.5	LUL	95.0

**Figure 1:**
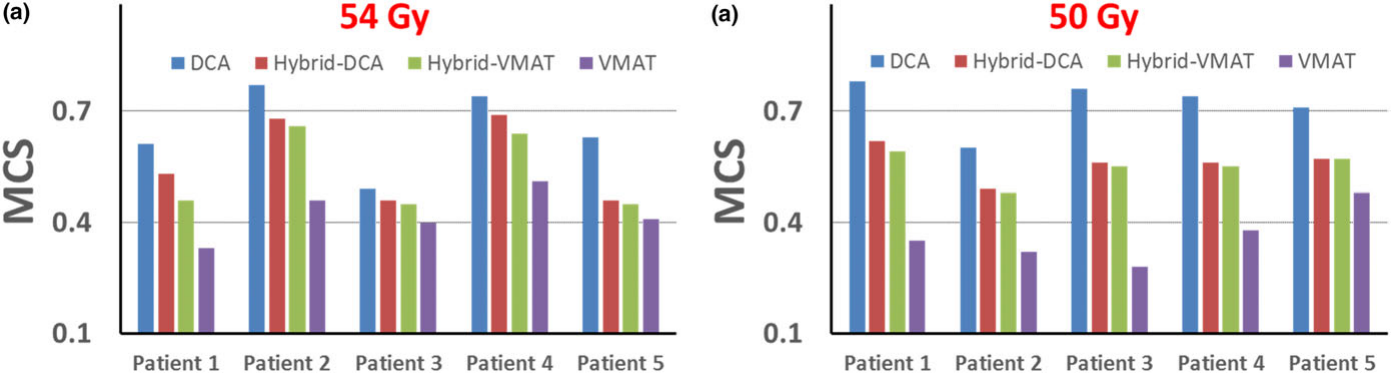
Comparisons of MCS values of four different planning strategies for two groups of SBRT treatment. (a) 54 Gy and (b) 50 Gy.

Table 3 shows the average and standard deviations of MCS, Total MUs, and gamma passing rates for all four planning techniques. Clear increases of the MCS value and gamma passing rate were observed with increasing DCA contributions for both patient groups. Total MUs used in the plans decreased as the DCA contributions were increased, indicating better delivery efficiency. The MCS values were compared among the four techniques for each individual patient, and the results show that MCS values decrease with increasing contribution of VMAT (Fig. 1). The Mann–Whitney *U* tests were performed to compare the evaluation metrics between different planning strategies. The resulting *P*‐values are listed in Table 4. No significant differences were found between Hybrid‐DCA and Hybrid‐VMAT techniques (*P* > 0.05). However, statistically significant differences for MCS were observed among DCA, Hybrid and VMAT strategies (*P* < 0.05). The plan complexities of the four planning strategies are related as follows: DCA < (Hybrid‐DCA ~ Hybrid‐VMAT) < VMAT. Pearson correlation tests were performed to evaluate the correlation among the MCS, total MUs and gamma passing rate. The correlation coefficients (*r*) between MCS and total MUs were −0.99 for 54 Gy patient group and −0.93 for 50 Gy patient group, respectively. This indicates a strong positive correlation between plan complexity and delivery efficiency (Appendix). The correlation coefficient between MCS and gamma passing rates (2%/2 mm threshold) were 0.97 and 0.86 for 54 Gy and 50 Gy patient groups, respectively. This indicates a strong negative correlation between the plan complexity and patient specific QA results.

**Table 3:** Comparisons of average MCS, total MUs and gamma passing rates for four different planning techniques

		MCS	Total MUs	*γ*(3%/3 mm)	*γ*(2%/2 mm)
54 Gy	DCA	0.65 ± 0.11	3446 ± 302	99.9 ± 0.2	98.0 ± 1.2
	Hybrid‐DCA	0.56 ± 0.12	4091 ± 488	99.6 ± 0.5	95.3 ± 2.8
	Hybrid‐VMAT	0.53 ± 0.11	4682 ± 1009	97.9 ± 1.9	93.3 ± 2.9
	VMAT	0.42 ± 0.07	5451 ± 1621	96.9 ± 2.5	91.6 ± 3.1
50 Gy	DCA	0.72 ± 0.07	1935 ± 179	99.3 ± 1.1	95.2 ± 3.5
	Hybrid‐DCA	0.55 ± 0.05	2270 ± 208	98.3 ± 1.6	91.6 ± 2.8
	Hybrid‐VMAT	0.55 ± 0.04	2663 ± 423	95.7 ± 2.4	87.3 ± 2.8
	VMAT	0.36 ± 0.08	2937 ± 437	93.9 ± 2.8	86.8 ± 3.2

**Table 4:** *P*‐values from the Mann–Whitney *U* test for MCS among four different planning techniques

	Hybrid‐DCA	Hybrid‐VMAT	VMAT
DCA	0.005	0.003	0.0001
Hybrid‐DCA	x	0.456	0.0007
Hybrid‐VMAT	x	x	0.0001

**Figure 2:**
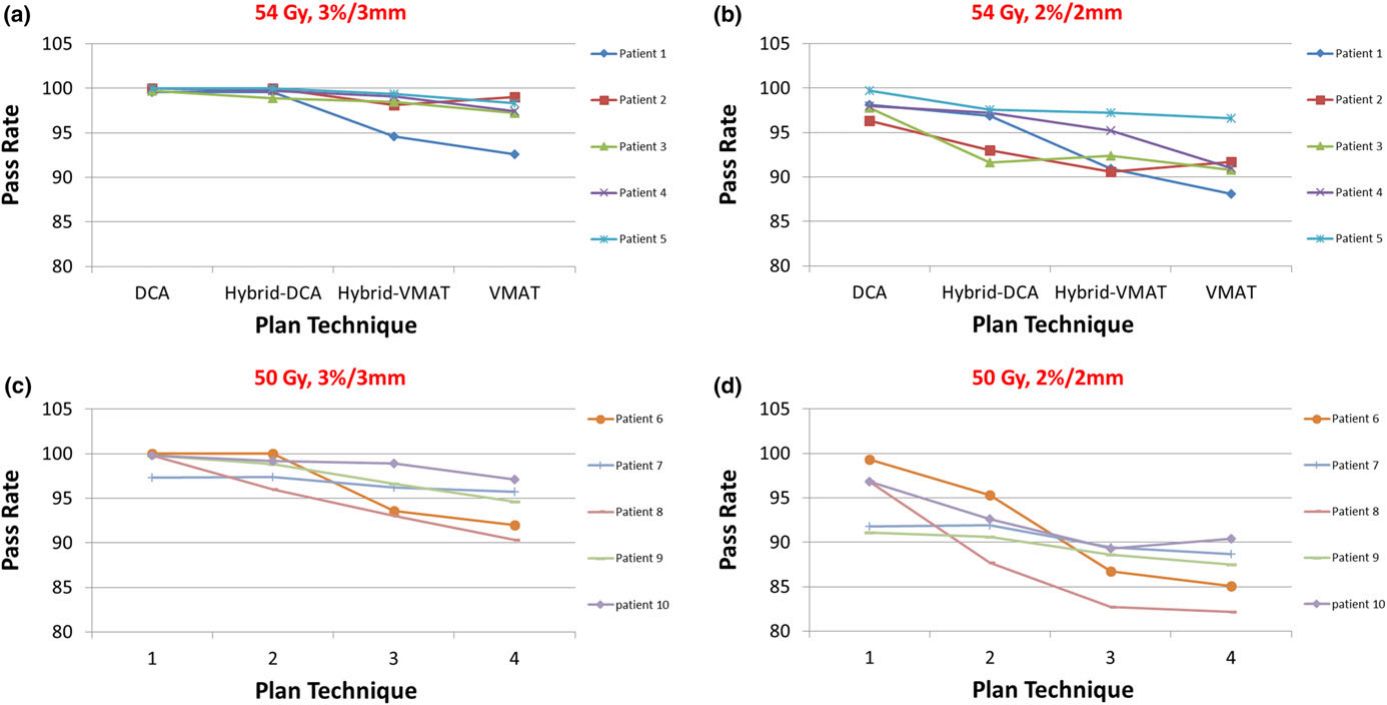
Comparisons of gamma passing rates of four planning strategies for two groups of SBRT treatment. (a) 3%/3 mm for 54 Gy; (b) 2%/2 mm for 54 Gy; (c) 3%/3 mm for 50 Gy; (d) 2%/2 mm for 50 Gy.

Figure 2 compares gamma passing rates of different strategies for both 3%/3 mm and 2%/2 mm thresholds. Gamma passing rates increase with higher DCA contributions from the plan, suggesting better agreement between the planned and delivered doses with higher DCA contributions.

**Figure 3:**
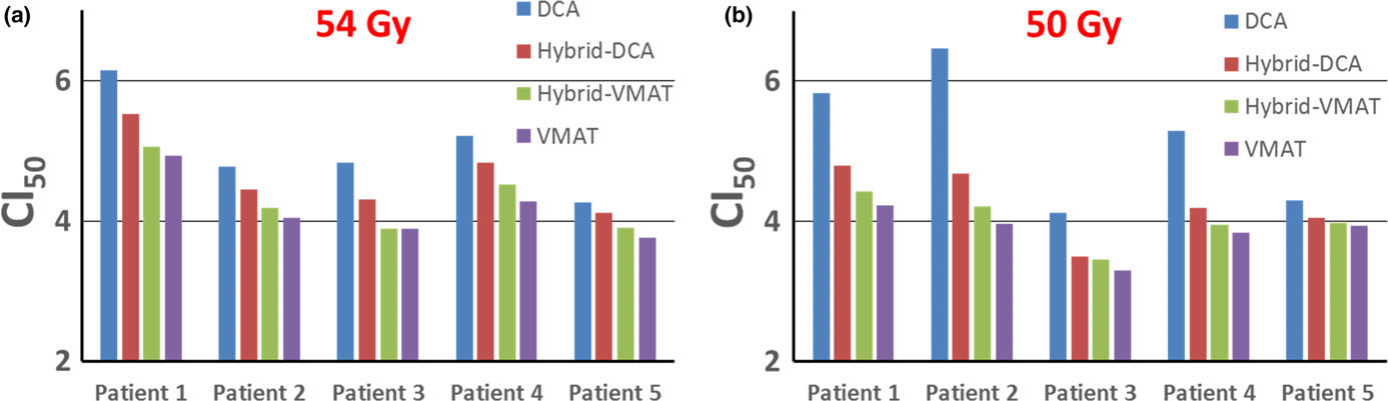
Comparisons of conformity index of four planning strategies for two groups of SBRT treatment. (a) 54 Gy and (b) 50 Gy.

As shown in Fig. 3, a clear improvement of *CI*_50_ was observed with higher VMAT contribution in treatment planning. This can be attributed to the increased capability of MLC modulation and dose shaping from the VMAT technique.

**Figure 4:**
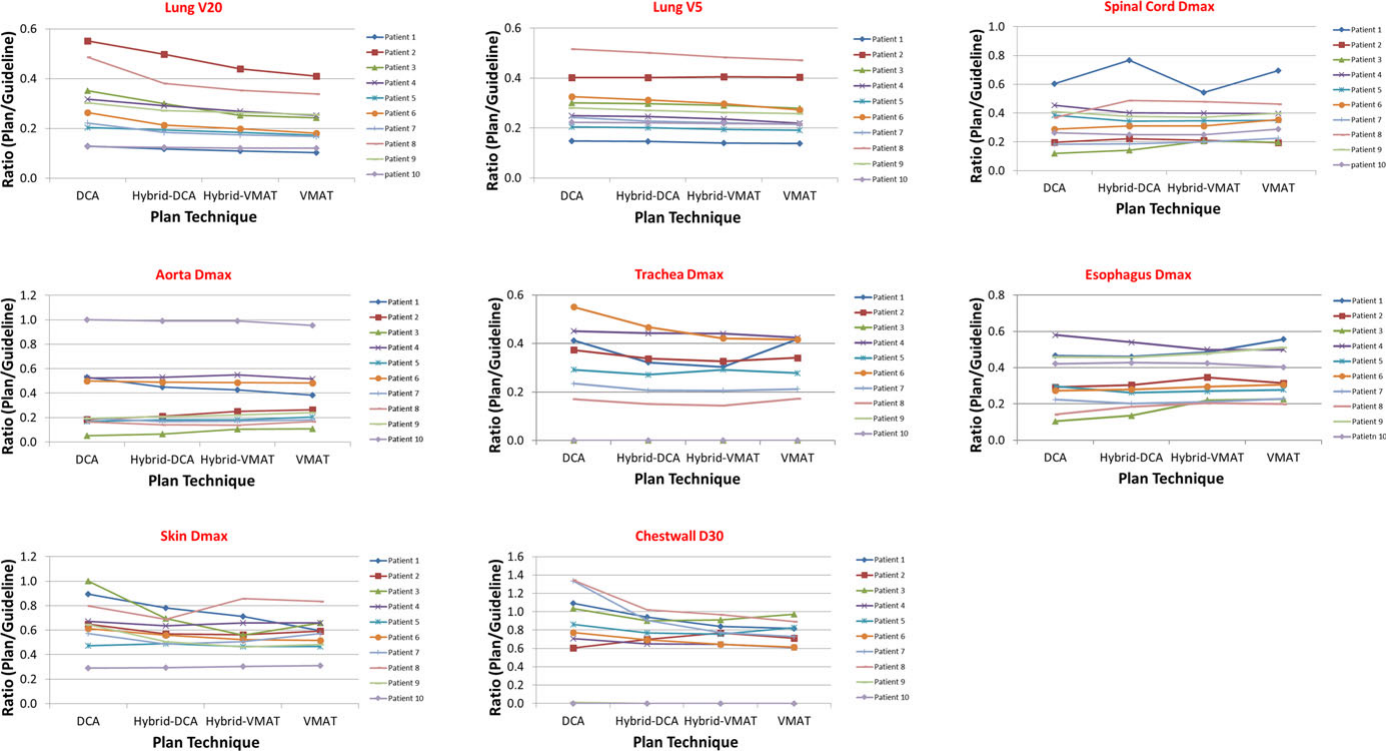
Comparisons of dosimetric index ratios (*V*_20_, *V*_5_, *D*_max_) of four planning techniques to the department guidelines for critical structures.

Figure 4 shows the dosimetric index ratios of selected critical structures between plans and our department guidelines for all four planning strategies. There were little variation between all four techniques with a few exceptions.

## 4 DISCUSSION

SBRT has become standard of care for the management of medically inoperable early stage non‐small‐cell lung cancer with superior treatment outcome compared to conventional radiotherapy techniques. Over the last decade SBRT planning techniques have evolved from static‐based beam configurations (3D‐CRT and IMRT) towards arc‐based (DCA and VMAT) configurations. Both DCA and VMAT techniques have their own pros and cons, and the superiority of one vs the other has been previously inconclusive. In this study we proposed a hybrid strategy which combines DCA and VMAT techniques. With certain fractions (1/3 or 2/3 in this study) of the prescription dose delivered by the DCA beam, uncertainties of the highly modulated VMAT plans are minimized, while VMAT contributions from the hybrid strategy allows for dose shaping around the target volume when the critical structure sparing is a concern. In this study the fractions of total prescription dose delivered by the DCA beams were chosen to be 1/3 and 2/3 as a demonstration. In practice, the planners can choose any other numbers they feel comfortable to develop the hybrid plan.

For 5 out of 10 patients studied (four 54 Gy patients and one 50 Gy patient), the DCA technique did not result in acceptable plan quality (based on our department guidelines), mainly due to the proximity of the target volume to the critical structures. This was improved by adding some VMAT components to the treatment plan, as Hybrid‐DCA produced only one unacceptable plan. With more VMAT contributions, Hybrid‐VMAT and VMAT produced no unacceptable plans. This finding is not surprising due to the better dose shaping capability of the VMAT compared to DCA. For both groups of patients, the conformity index improved with increasing VMAT usage, as was the dose sparing to critical structures (Fig. 3). Compared to the VMAT technique, a total MU reduction of 14%, 25% and 37% were found for Hybrid‐VMAT, Hybrid‐DCA and DCA techniques for 54 Gy patient group, and 9%, 23% and 34% for 50 Gy patient group. If we assume a dose rate of 1400 MU/min for both DCA and VMAT beams, that was corresponding to beam on time reduction of 33, 57 and 85 s for Hybrid‐VMAT, Hybrid‐DCA and DCA techniques for 54 Gy patient group, and 12, 29 and 42 s for 50 Gy patient group. This finding indicates a significant improvement in the delivery efficiency with increasing usage of DCA, which could result in less intra‐fraction motion and increased patient comfort during treatment.

In this study, the overall plan complexity was evaluated by a single metric, MCS (between 0 and 1), for all four planning strategies. MCS incorporates the leaf sequence variability and aperture area variability into the calculation. As seen in Table 3 and Fig. 1, an increase in MCS was found with more contribution from DCA. We did not observe significant variations of plan complexity between the hybrid‐DCA and hybrid‐VMAT techniques (*P* > 0.05 from the Mann–Whitney *U* test). However, statistically significant differences in MCS values were found among DCA, Hybrid and VMAT strategies (*P* < 0.05), which indicates adding DCA components in the VMAT plan can reduce the plan complexity and increase the plan deliverability.

In our clinical practice, the guideline for passing the patient specific QA for SBRT is *γ* > 95% with 3%/3 mm threshold. Better agreement between the planned and delivered doses were found with more DCA usage in the treatment plans (the average *γ* were 96.6%, 97.9%, 99.6% and 99.9% for VMAT, Hybrid‐VMAT, Hybrid‐DCA and DCA for 54 Gy patient group, and 93.9%, 95.7%, 98.2% and 99.3% for 50 Gy patient group). Results from tighter criteria (2%/2 mm) were also included in this analysis to gain more insight of the deviation between the planned and delivered doses as the DCA/VMAT contribution varies. Better QA results for plans with more DCA contributions can be attributed to reduced plan modulation and less plan complexity. The 54 Gy patient group had better QA results than 50 Gy group partially due to patient selection for different prescription dose. The 50 Gy prescription was selected for patient treatment if there was a critical structure dose concern, which is a reason of increased MLC modulation in the plan.

In our clinic, VMAT is the treatment choice due to its capability for dose shaping when critical organ sparing becomes important. By incorporating DCA component into VMAT planning, the hybrid technique is favored over VMAT technique because it offers comparable dosimetry to VMAT, while increasing the delivery efficiency, minimizing the MLC complexity, and increase the agreement between the planned and delivered doses.

## 5 CONCLUSION

This study focuses on possible dosimetric and delivery efficiency advantages of hybrid treatment planning strategy combining DCA and VMAT beams for lung SBRT treatment. By adding partial DCA components to the VMAT plans, we demonstrated that the hybrid plans result in better plan conformity, less plan complexity and better agreement between the planned and delivered doses. Furthermore, the more DCA contribution in the plan, the less MUs used and the better the delivery efficiency. The improvement of beam on time may reduce the uncertainty due to patient intra‐fraction motion and also increase the patient comfort during the course of treatment.

## CONFLICT OF INTEREST

The authors declare no conflict of interest.

## APPENDIX 1

### DEFINITION OF MODULATION METRICS (MCS)

The MSC for VMAT plans was originally defined by McNiven for step‐and‐shoot IMRT plans,^14^ and then modified by Masi in order to apply for VMAT plans.^15^ The LSV is defined based on the difference in position for adjacent MLC leaves in the same bank for each control point (CP). The positional variations are considered relative to the maximum MLC variation per bank for each CP. The maximum variation per CP is defined as:

$${\mathrm{Pos}}_{\max}\left(CP\right)={\left\langle \text{max}\left({\mathrm{pos}}_{n\in N}\right)-\text{min}\left({\mathrm{pos}}_{n\in N}\right)\right\rangle }_{\mathrm{leaf}\;\mathrm{bank}},$$

$$\begin{array}{cc} LSV_{\mathrm{CP}}= & {\left(\frac{\sum_{n\;=\;1}^{N\;-\;1}(pos_{\max}-|pos_{n}-pos_{n\;+\;1}\left|\right)}{\left(N-1\right)\;\times \;pos_{\max}}\right)}_{left\;bank}\\ & \times {\left(\frac{\sum_{n\;=\;1}^{N\;-\;1}(pos_{\max}-|pos_{n}-pos_{n\;+\;1}\left|\right)}{\left(N-1\right)\;\times \;pos_{\max}}\right)}_{right\;bank}, \end{array}$$

where *N* is the number of moving leaves and *pos*_*n*_ is the position of leaf *n*.

The AAV is based on the area defined by opposing MLC leaves in a CP normalized to the maximum area in the arc, which is defined by the maximum apertures for all leaf pairs over all CPs in the arc

$$\frac{{\mathrm{AAV}}_{\mathrm{CP}}=\sum_{a=1}^{A}\left({\left\langle pos_{a}\right\rangle }_{left\;bank}-{\left\langle pos_{a}\right\rangle }_{right\;bank}\right)}{\sum_{a\;=\;1}^{A}\left({\left\langle \text{max}\left(pos_{a}\right)\right\rangle }_{left\;bank\;\in \;arc}-{\left\langle \text{max}\left(pos_{a}\right)\right\rangle }_{right\;bank\;\in \;arc}\right),}$$

where *A* is the number of moving leaves in the arc.

The MLCs are continuously moving between CP while MU are being delivered, so MCS considers the mean values of *LSV*_*CP*_ and *AAV*_*CP*_ between adjacent CPs. This product is weighted by the percentage of total MU delivered between the adjacent CPs:

$$MCS=\sum_{i=1}^{I-1}\left[\frac{\left({\mathrm{AAV}}_{CP_{i}}+AAV_{CP_{i+1}}\right)}{2}\times \frac{\left({\mathrm{LSV}}_{CP_{i}}+LSV_{CP_{i+1}}\right)}{2}\times \frac{{\mathrm{MU}}_{CP_{i,i+1}}}{MU_{\mathrm{arc}}}\right],$$

where I is the number of CP and MU_CPi,i+1_ are the MU delivered between two consecutive CPs.

The *AA* term is defined using the notation from above as

$$AACP=\sum_{a=1}^{A}\left({\left\langle {\mathrm{pos}}_{a}\right\rangle }_{left\;bank}-{\left\langle pos_{a}\right\rangle }_{right\;bank}\right)\times w_{a}$$

where *w*_*a*_ is the width of leaf *a*

$$AA_{\mathrm{arc}}=\frac{\sum_{i=1}^{I-1}\left[\frac{\left({\mathrm{AACP}}_{CP_{i}}+AACP_{CP_{i+1}}\right)}{2}\times \frac{MU_{CP_{i,i+1}}}{MU_{\mathrm{arc}}}\right]}{I-1}.$$
